# HLA and killer cell immunoglobulin-like receptor (KIRs) genotyping in patients with acute viral encephalitis

**DOI:** 10.18632/oncotarget.24778

**Published:** 2018-04-03

**Authors:** Antonino Tuttolomondo, Claudia Colomba, Danilo Di Bona, Alessandra Casuccio, Domenico Di Raimondo, Giuseppe Clemente, Valentina Arnao, Rosaria Pecoraro, Paolo Ragonese, Anna Aiello, Giulia Accardi, Rosario Maugeri, Carlo Maida, Irene Simonetta, Vittoriano Della Corte, Domenico Gerardo Iacopino, Calogero Caruso, Antonio Cascio, Antonio Pinto, on behalf of KIRIIND (KIR Infectious and Inflammatory Diseases) Collaborative Group

**Affiliations:** ^1^ U.O.C di Medicina Interna con Stroke Care, Dipartimento Biomedico di Medicina Interna e Specialistica (Di.Bi.M.I.S), Università degli Studi di Palermo, Palermo, Italy; ^2^ U.O.C di Malattie Infettive, Dipartimento di Scienze per la Promozione della Salute e Materno Infantile G. D'Alessandro, Università degli Studi di Palermo, Palermo, Italy; ^3^ Dipartimento di Biopatologia e Biotecnologie Mediche, Università degli Studi di Palermo, Palermo, Italy; ^4^ Dipartimento di Scienze per la Promozione della Salute e Materno Infantile “G. D'Alessandro”, Università degli Studi di Palermo, Palermo, Italy; ^5^ Dipartimento di BioMedicina Sperimentale e Neuroscienze Cliniche, Università degli Studi di Palermo, Palermo, Italy; ^6^ School of Allergology, Dipartimento delle Emergenze e Trapianti d'Organo, University of Bari, Bari, Italy

**Keywords:** KIRs, HLA, encephalitis

## Abstract

**Introduction:**

The HLA genes, as well as the innate immune KIR genes, are considered relevant determinants of viral outcomes but no study, to our knowledge, has evaluated their role in the clinical setting of acute viral encephalitis.

**Results:**

Subjects with acute viral encephalitis in comparison to subjects without acute viral encephalitis showed a significantly higher frequency of 2DL1 KIR gene and AA KIR haplotypes and of HLA-C2 and HLA-A-Bw4 alleles. Subjects without acute viral encephalitis showed a higher frequency of interaction between KIR2DL2 and HLAC1. Multiple logistic regression analysis showed the detrimental effect of HLA-A haplotype and HLA-C1, HLA-A-BW4 HLA-B-BW4^T^ alleles, whereas multiple logistic regression showed a protective effect of AB+BB KIR haplotype and a detrimental effect of interaction between KIR3DL1 and HLA-A-Bw4.

**Discussion:**

Our findings of a lower frequency of activating receptors in patients with acute encephalitis compared to controls could result in a less efficient response of NK cells. This finding could represent a possible pathogenetic explanation of susceptibility to acute symptomatic encephalitis in patients with viral infection from potentially responsible viruses such as Herpes virus.

**Materials and Methods:**

30 Consecutive patients with symptomatic acute viral encephalitis and as controls, 36 consecutive subjects without acute encephalitis were analyzed. The following KIR genes were analyzed, KIR2DL1, 2DL2, 2DL3, 2DL5, 3DL1, 3DL2, 3DL3, 2DL4, 2DS1, 2DS2, 2DS3, 2DS4, 2DS5, 3DS1, 2 pseudogenes (2DP1 and 3DP1) and the common variants of KIR2DL5 (KIR2DL5A, KIR2DL5B).

## INTRODUCTION

Acute encephalitis is a severe form of neurological disease caused by inflammation of the brain parenchyma. It is commonly characterized by the acute onset of fever, altered mental status, new onset of focal neurological symptoms, and generalized or focal seizures. It can be produced by various etiologies, but viral infection and autoimmune disorders are the most common [1, 2]. Herpes simplex encephalitis (HSE) is a complication of herpes simplex virus (HSV) infection of the central nervous system (CNS) [3].

Natural killer (NK) cell activation or inhibition pathways mediated by HLA expression could have a potential detrimental or protective role in viral encephalitis onset in immunocompetent hosts [4–10]. NK cell target recognition is strictly linked to the role of human leukocyte antigen (HLA) class I molecules and of killer immunoglobulin-like receptors (KIRs), activating or inhibitory receptors on the surface of NK cells and T-cells [11].

Two different haplotypes of KIR genes have been reported. The A haplotype with a activating KIR gene, KIR2DS4, and five inhibitory KIR genes (KIR2DL1, KIR2DL3, KIR3DL1, KIR3DL2, and KIR3DL3), whereas the B haplotype has a variable numbers of activating and inhibitory genes [12]. KIRs act by binding to specific alleles of HLA-C, HLA-B, or HLA-A [13].

Our group previously reported [14] that immunocompetent subjects carrying the homozygous A haplotype or the HLABw4T allele are at higher risk of developing symptomatic disease after primary cytomegalovirus (CMV) infection.

The HLA genes, as well as the innate immune KIR genes, are considered relevant determinants of viral outcomes; however, their interaction with each other has not been fully studied in viral infections, and no study, to the best of our knowledge, has evaluated their role in the clinical setting of acute viral encephalitis.

### Study hypothesis

The hypothesis of our study was that a higher risk of developing symptomatic encephalitis due to herpes viruses may be related to prevalence of some KIR genes and HLA-ligand alleles and of their interactions.

### Aim of the study

The aim of our study was to apply knowledge about the immunological background of acute viral encephalitis in connection with the frequency of the KIR genes and HLA-ligand alleles in a small sample of patients and thereby contribute to the definition of a possible susceptibility profile of symptomatic viral encephalitis.

## RESULTS

We enrolled 30 patients referred by the wards of Internal Medicine and Stroke Care, Infectious Diseases, and Neurology of the “Paolo Giaccone” University Hospital of Palermo and that have been diagnosed as suffering from acute viral encephalitis, and 36 healthy subjects without acute encephalitis .

All the enrolled patients (case and controls) were of Caucasian ethnicity, and they were admitted from the city of Palermo and from the province of Palermo, which is the most populous city in Sicily (Italy).

General and clinical characteristics of patients with acute encephalitis and subjects without acute encephalitis are listed in Table 1. Mean age of patients with acute viral encephalitis was 67.53 ± 7.59 years in subjects with encephalitis and 72.53 ± 12.62 years in subjects without encephalitis.

**Table 1:** General, demographic and clinical findings in subjects with acute viral encephalitis and in healthy subjects

Variables	Subjects with acute encephalitis (*n*: 30)	Subjects without acute encephalitis (*n*: 36)	*p*
**Sex (M/F) (*n*%)**	12/18 (40/60)	22/14 (61.1/38.8)	0.13
**Age ( years) (mean ± sd)**	67.53 ± 7.59	72,53 ± 12.62	0.06
**Diabetes (*n*/%)**	9 (30)	8 (22.2)	0.57
**Hypertension (%/*n*)**	10 (30)	10 (27.7)	0.78
**Previous cardiovascular events**	12 (40)	16 (44.4)	0.89
**WBC (× 10**^3^**/μL ) ( mean ± sd)**	10.480 ± 2.24	8.20 ± 1.79	**<0.0005**
**Neutrofils (%)(mean ± sd)**	65.07 ± 12.6	71,50 ± 7.1	**0,011**
**Lymphocyte (%) ( mean ± ds)**	29.93 ± 12.06	19.6 ± 5.01	**<0.0005**
**RBC (× 10**^6^**/µL ) ( mean ± ds)**	5.26 ± 5.9	4.25 ± 1.09	0.31
**Haemoglobin (g/dL) (mean ± sd)**	11,69 ± 2.19	12,46 ± 0.61	**0.048**
**ESR ( mm/h) (mean ± sd)**	42,77 ± 14.13	25,47 ± 14.74	**<0.0005**
**RCP (mg/dL) (mean ± sd)**			
**Determined viral etiology**	30 (100%)	-	
**positivity of viral PCR in CRF**			
**HSV-1**	29 (96.6)		
**HHV- 6**	1 (3.34)		
**CSF findings(%/*n*)**			
**Normal**	8 (26.6)		
**Pleiocitosis**	12 (40)		
**Lymphocyte**	10 ( 33.3)		
**protein mild**	24 (80)		
**normal glucose**	28 (93.3)		
**MRI findings(%/*n*)**			
**Positive**	20 (66,6)		
**Negative**	6 (16.2)		
**Not performed**	4 (10,8)		
**EEG findings(%/*n*)**			
**Positive**	12 (40)		
**Negative**	2 (13.3)		
**Not performed**	16 (53.3)		

ESR: erythrocyte sedimentation rate; RCP: C-reactive protein; WBC: white body cell; RBC: red body cell; HSV: herpes virus; HHV -6: Human herpesvirus 6; HSV-1: herpes simplex virus type 1; CSF: cerebrospinal fluid; MRI : Magnetic Resonance Imaging; EEG: Electroencephalography.

In comparison to subjects without encephalitis subjects with encephalitis showed significantly higher WBC (1048 ± 2.24 vs. 8.20 ± 1.79; *p <* 0.0005) and lymphocyte percentage (29.93 ± 12.06 vs. 19.6 ± 5.01; *p <* 0.0005). A viral etiology was found in 30 (100%) cases. The molecular biological investigations performed on the CSF were documented positive by means PCR for human herpes virus 1 DNA (HSV-DNA) (29 cases), human herpes virus-6 (HHV type 6) (1 case) .

The chemical-physical examination of cerebrospinal fluid documented these CSF findings: normal in 4 (13.3%) cases, protidorrachia was mildly increased in 24 (80%) cases, mild pleocytosis was observed in 25 (83.3%) cases, while in the remaining 5 (16.6%) CSF samples was absent (Table 1).

The KIR analysis of patients with acute encephalitis and of subjects without acute encephalitis (see Table 2) showed the ubiquitous presence of genes coding for 2DL3, 2DL4, 3DL2 and 3DL3, and of 2DP1, 3DP1 (100% of patients).

**Table 2:** Frequencies of KIR genes and HLA allele and haplotypes among individuals with symptomatic acute encephalitis and healthy subjects

KIR haplotypes	Subjects with acute encephalitis (*n*: 30)	Subjects without acute encephalitis (*n*: 36)	*p*	
**AA** **AB + BB**	19 (63.3) 11 (36.7)	8 (22.2) 28 (77.8)	**0.001**	
**KIR alleles**				
**2DL1 (*n*/%)**	30 (100)	31 (86.1)		**0.034**
**2DL2 (*n*/%)**	12 (40)	18 (50)		0.41
**2DL3 (*n*/%)**	30 (100)	36 (100)		
**2DL4 (*n*/%)**	30 (100)	36 (100)		
**2DL5A (*n*/%)**	6 (20)	6 (16.6)		0.72
**2DL5B (*n*/%)**	3 (10)	6 (16.6)		0.43
**2DS1 (*n*/%)**	9 (30)	10 (27.7)		0.84
**2DS2 (*n*/%)**	5 (16.6)	12 (33.3)		0.12
**2DS3 (*n*/%)**	3 (10)	8 (22.2)		0.18
**2DS4 (*n*/%)**	19 (63.3%)	32 (88.8)		**0.011**
**2DS5 (*n*/%)**	6 (20)	19 (52.7)		**0.006**
**3DL1 (*n*/%)**	30 (100)	32 (88.8)		0.060
**3DL2 (*n*/%)**	30 (100)	36 (100)		-
**3DL3 (*n*/%)**	30 (100)	36 (100)		-
**3DS1 (*n*/%)**	6 (20)	22 (61.1)		**0.001**
**2DP1 (*n*/%)**	30 (100)	36 (100)		-
**3DP1 (*n*/%)**	30 (100)	36 (100)		-
**HLA-A-Bw4 (*n*/%)**	12 (40)	3 (8.3)		**0.003**
**HLA-B-Bw4**^T^ **(*n*/%)**	13 (43.3)	8 (22.2)		0.067
**HLA-B-Bw4**^I^ **(*n*/%)**	5 (16.6)	7 (19.4)		1.0
**HLA- C1 (*n*/%)**	21 (70)	22 (61.1)		0.45
**HLA- C2 (*n*/%)**	23 (76.6)	13 (36.1)		**0.001**

KIR: Killer-cell immunoglobulin-like receptors; HLA: human leukocyte antigen.

In comparison to control subjects subjects with acute viral encephalitis showed a significantly higher frequency of KIR 2DL1 gene (100% vs. 86.1% *p =* 0.034)

In comparison to controls patients with acute viral encephalitis also showed a higher frequency AA KIR haplotype (63.3% vs. 22.2%; *p =* 0.001), HLA-C2 (76.6% vs. 36.1%; *p <* 0.001) and of HLA-A-Bw4 (40% vs. 8.3%; *p =* 0.003 ) alleles.

With regard to KIR genes, in comparison to subjects with acute viral encephalitis subjects without encephalitis showed respectively a higher frequency of 2DS4 (88.8% vs. 63.3%; *p =* 0.011); 2DS5 (52.7% vs. 20%; *p =* 0.006), and 3DS1 (61.1% vs. 20%; *p <* 0.001). With regard to interaction between HLA-ligand alleles and KIR genes, subjects with acute viral encephalitis showed no significant difference in frequency of co-expression of any considered HLA and KIR, whereas subjects without acute viral encephalitis had a higher frequency of interaction between KIR2DL2 and HLA-C1 ( 50% vs. 16.6%; *p =* 0.028) (See Table 3).

**Table 3:** Frequencies of KIR-HLA combinations among individuals with symptomatic acute encephalitis and healthy subjects

KIR and HLA combinations	Subjects with acute encephalitis (*n*: 30)		Subjects without acute encephalitis (*n*: 36)	*p*
**2DL 2-HLA-C1 (*n*/%)**	5 (16.6)	15 (50)		0.028
**2DL 3-HLA-C1 (*n*/%)**	4 (13.3)		12 (33.3)	0.059
**2DS2-HLA-C1 (*n*/%)**	4 (13.3)		11 (30.5)	0.096
**2DS2-HLA-C2 (*n*/%)**	1 (3.3)		2 (5.5)	0.66
**2DL2- HLA-C2 (*n*/%)**	4 (13.3)		4 (11.1)	0.78
**2DS1-HLA-C2 (*n*/%)**	5 (16.6)		4 (11.1)	0.51
**2DL1-HLA-C1 (*n*/%)**	6 (20)		3 (8.3)	0.16
**2DL1-HLA-C2 (*n*/%)**	3 (10)		4 (11.1)	0.88
**3DL1-HLA-Bw4**^T^ **(*n*/%)**	9 (30)		4 (11.1)	0.055
**3DS1-HLA-Bw4**^T^ **(n/%)**	4 (13.3)		6 (16.6)	0.70
**3DS1-HLA-A-BW4 (*n*/%)**	4 (13.3)		6 (16.6)	0.707
**3DS1-HLA-B-BW4**^T^ **(*n*/%)**	4 (13.3)		6 (16.6)	0.707
**3DS1-HLA-B-BW4**^I^ **(*n*/%)**	3 (10)		4 (11.1)	1.0
**3DL1-HLA-A-BW4 (*n*/%)**	4 (13.3)		3 (8.3)	0.693
**3 DL1-HLA-B-BW4**^T^ **(*n*/%)**	9 (30)		4 (11.1)	0.055
**3DL1-HLA-B-BW4**^I^ **(*n*/%)**	4 (13.3)		3 (8.3)	0.693

KIR: Killer-cell immunoglobulin-like receptors; HLA: human leukocyte antigen.

The multiple logistic regression analysis considering variables predictive of the occurrence of acute viral encephalitis showed the detrimental effect of AA KIR haplotype [Exp (β): 8.38; 95% CI: 1.27–55.45; *p =* 0.027], HLA-C1[Exp (β): 8.92; 95% CI: 1.04–76.2; *p =* 0.046], HLA-A-BW4 [Exp (β): 36.7; 95% CI 3.51–382.4; *p =* 0.003] and HLA-B-BW4^T^ [Exp (β): 7.9; 95% CI 1.14–56.11; *p =* 0.037] alleles (See Table 4).

**Table 4:** Logistic regression model to predict the occurrence of symptomatic acute encephalitis

Variable	Exp (β)	95% Confidence interval for exp(B)	*P* Value
***Kir Haplotype***			
**AA**	8.38	1.27–55.45	**0.027**
***HLA alleles***			
**HLA- C1**	8.92	1.04–76.2	**0.046**
**HLA-A-Bw4**	36.7	3.51–382.4	**0.003**
**HLA-B-BW4**^T^	7.98	1.14–56.11	**0.037**
***Interaction KIR and HLA haplotype***			
**2DL2-HLA-C1**	0.080	0.007–0.86	**0.037**
**2DL3-HLA-C1**	0.032	0.003–0.34	**0.004**
**2DS2-HLA-C1**	0.096	0.01–0.72	**0.023**
**3DL1-HLA-A-BW4**	43.04	2.57–720.0	**0.009**

Furthermore, with regard the KIR-HLA group ligand interactions, multiple regression analysis showed a detrimental effect of interaction between KIR 3DL1 and HLA-A-Bw4 alleles [Exp (β): 43.04; 95% CI 2.57–720.0; *p =* 0.009] and a protective effect of interaction between KIR2DL2 and HLA-C1 [Exp (β): 0.080; 95% CI 0.007–0.86; *p =* 0.037], KIR 2DL3 and HLA-C1 [Exp (β): 0.032; 95% CI 0.003–0.34; *p =* 0.004] and KIR2DS2 and HLA-C1 [Exp (β): 0.096; 95% CI 0.01–0.72; *p =* 0.023] (See Table 4).

## DISCUSSION

Our study shows that in immunocompetent adult subjects there is an association between some KIR genes and HLA-ligand alleles and susceptibility to develop a symptomatic acute viral encephalitis. Considering the complexity of genetic studies that have led to the identification of a few candidate genes and related only to some viruses (herpes viruses, West Nile virus, and viruses transmitted by ticks), we hypothesized that by contributing of the high polymorphism of KIR to the variability of both the innate and adaptive immune response, these glycoproteins could be the subject of study for the disease.

Definition of the genetic and immunological background of acute viral encephalitis can play a key role to determine personalized medicine. In this sense, our goal was to try to outline the immunological background of the disease in relation to the genetic analysis of KIRs and HLA alleles, with the aim of implementing the knowledge and treatment.

Our findings show that subjects with acute viral encephalitis are more likely to have a AA KIR haplotype. Thus it appears conceivable to hypothesize that AA KIR haplotype carrier subjects are more susceptible to the development of the disease. A previous study [14] had already demonstrated a statistically significant association between the AA haplotype and the negative effects on the course of CMV infection in immunocompetent individuals.

Few studies have analyzed the relationship between HLA haplotype and encephalitis subsceptibility. Lebon *et al*. did not establish any association between susceptibility to measles encephalitis and HLA markers in 24 patients vs. controls in 1926 [22].

Our finding of an association between the AA KIR haplotype and development of acute encephalitis is a novel finding in the setting of acute viral encephalitis, but it is consistent with findings from other studies regarding a higher rate of CMV reactivation in the bone marrow transplantation clinical setting in subjects carrying AA KIR haplotype [17], whereas in a further study in the setting of kidney transplantation, the rate of CMV reactivation was inversely correlated with the number of activating KIRs [18].

The frequency of AA haplotype in Italy is 26% and in Europe it varies from 12% to 38%, on average about 30% [19], its frequency in our control subjects is 22%. Thus, it is consistent with that reported in the literature, whereas AA haplotype frequency of 56.6% in subjects with acute symptomatic viral encephalitis indicate an over-representation of this weak haplotype in subjects with acute encephalitis also confirming that our control subjects are adequate and representative of the general Italian population.

Our findings of a lower frequency of activating KIR genes in patients with acute encephalitis compared to controls could result in a less efficient response of NK cells.

Despite the small number of enrolled patients, since viral encephalitis is a rare disease our findings could corroborate a possible speculation about the role of NK cell activation or inhibition pathways mediated by HLA expression and their interaction with killer immunoglobulin-like receptors (KIRs) on the surface of NK cells and T-cells

Concerning HLA-allele frequency in subjects with acute viral encephalitis in comparison to subjects without acute viral encephalitis our findings show a higher frequency of HLA-C2, HLA-A- Bw4 and HLA-B-BW4^T^_._

Group 1 HLA-C (HLA-C1) allotypes have an asparagine residue at position 80 that is recognized by KIR2DL2 and KIR2DL3, whereas group 2 HLA-C(HLA-C2) allotypes by means position 80 lysine, are recognized by KIR2DL1 [15]. Furthermore, KIR2DS1 has reported as able to bind to HLA-C2 allotypes [16], whereas KIR2DS2 to HLA-C1 [17–23].

A recent study [24] analyzed whether HLA-C and KIR genotypes were associated with treatment outcome for chronic hepatitis B infection (CHB) showing that the combination of KIR2DL1 with its ligand HLA-C2 is predictive of clinical outcome of infection predicted. Other authors [17] also found that the KIR2DS3 - HLA-C2 interaction is predictive of viral persistence.

Thus, the HLA-allele and its interaction with the KIR haplotype may predict viral infection outcome by means of their effects on NK cell pathways. Consistent with this issue in a recent study [18] higher interferon (IFN)-γ production was observed within NK cells expressing KIR2DL1.

Notably, in our subjects with acute viral encephalitis the HLA-A-Bw4 was found to be a predictor of the risk of acute viral encephalitis by multivariate analysis (Exp (β): 9.53; *p =* 0.015) suggesting the importance of an interaction with other significant variables, such as the 3DL1 gene . These data suggest that possible interaction of HLA-Bw4 with the inhibitory receptor KIR3DL1 may counteract the activating receptors of the B haplotype in patients with acute viral encephalitis and this issue is further confirmed by the significant association at multivariate analysis of the interaction between KIR 3DL1 and HLA-A-BW4 allele and encephalitis (Exp (β): 43.04; *p =* 0.009).

To the best of our knowledge, this is the first study to show that the KIR-ligand group HLA-A-Bw4 can influence susceptibility to viral encephalitis. This effect may be mediated by the activation of the inhibitory KIR3DL1 [23], although an association with other HLA-B-Bw4 alleles [24] that bind the same KIR gene was not reported in this study. This may imply different binding affinity, and consequently, inhibitory ability between the HLA-A-Bw4 and HLA-B-Bw4 alleles. Furthermore, KIR3DL1 is among the most polymorphic of the KIR loci, and KIR3DL1 alleles show high variability in the level of their expression on cell surfaces, with functional repercussions [25]. Whatever the mechanism, a clear correlation between HLA-A-Bw4 alleles and susceptibility to encephalitis was reported in our study, pointing out the need for further investigations also on the variability of this KIR gene, aiming to improve the predictive value of the possible combined variable KIR3DL1/HLA-A-Bw4.

With regard to HLA-B-BW4^T^ this is the first study reporting a detrimental effect of this allele in the acute viral infection clinical setting. The rarity and highly sporadic nature of encephalitis offers some challenges mainly related to the definition of a susceptibility to the disease profile and diagnostic and therapeutic strategies. There are clear reports of host factors that are influential in determining viral outcome. The cellular immune response coordinated by CD4 and CD8 T cells appears to be important in this process.

Nevertheless, several studies [26–32] described how the association between different HLA–KIR combinations involving activating KIRs or inhibitory KIRs to different extents, can modify progression and prognosis of several infectious diseases by means a possible influence on T cells function [33–35].

Even in the context of acute viral encephalitis, herpes simplex virus type 1 infection has been reported as able to induce an immune response by means the activation of pattern recognition receptors and type I interferon production driving a adaptive immune response to initial viral infection [36–37]. Thus owing to the fact that many studies are beginning to implicate the immune response to HSV-1 and its various cell populations (e.g. microglia, CD8+ T cells) in causing widespread CNS pathology such as acute encephalitis, our finding of a higher frequency of some KIR haplotype (AA haplotype) and of some HLA allele such as HLA-C2 or HLA-A-BW4 may represent, to the best of our knowledge, the first report offering promising information about the immunologic background of patients with acute viral encephalitis.

Individual variations in immune status and function determine responses to infection and contribute to disease severity and outcome. Control of Central Nervous System (CNS) viral infections by the immune system is multifactorial, including viral recognition receptors (Toll-like receptors [TLRs] and RIG-I-like receptors [RLRs]), control of the permeability of the blood-brain barrier, and both innate and adaptive immune mechanisms. Certain HLA types, chemokines, and interferon pathway elements are associated with a risk of more severe outcomes in humans, and multiple pathways have been investigated in murine models. Our findings are consistent with this issue, reporting that some KIR haplotype and HLA alleles and their interaction are more frequent in subjects with acute viral encephalitis and suggesting that this immunological background may influence reactivation of an earlier infection in the majority of cases and neuroinflammatory process subsequent to viral infection driving to symptomatic encephalitis with symptoms that can include headache and fever personality and behavioral changes, seizures, hallucinations, and altered levels of consciousness

Several repertoire and complex and dynamic nature of innate immune cell functions such as those mediated by KIR/HLA interaction makes it likely that the neuroinflammatory response participates simultaneously in pathogenic, protective, and reparative aspects. On this basis it is challenging to consider future studies addressing the manipulation of immune cell infiltration or function as a therapeutic strategy for reducing CNS immunopathology, particularly within the context of viral encephalitis and other diseases with a immunoinflammatory pathogenesis of neuronal damage [38–42].

### Limitations

The main limitation of this study lies in its cross-sectional nature, making it impossible to dissect the temporal relation between genetic background and progression of encephalitis process.

Another limitation is small number of patients included in the analysis, nevertheless encephalitis is a nota frequent disease and our results could be of interest and support the issue that the KIR AA haplotype is disadvantageous for control of viral infections

Another limitation is due to the fact that T cells variably express receptors of the killer immunoglobulin-like receptor (KIR) family and we do not performed T-Cell Cloning and KIR Phenotyping of T-Cell Clones on Total RNA extracted from T-cell clones to evaluate the real burden of KIR receptors on T-Cells.

## CONCLUSIONS

This study reports that subjects with acute symptomatic encephalitis show in comparison to healthy controls:

1) a higher frequency of 2DL1 KIR gene (100% vs. 86.1% *p =* 0.034);

2) a higher frequency AA KIR haplotype

3) higher frequency of HLA-C2 and of HLA-A-Bw4 alleles.

Whereas control subjects without encephalitis showed in comparison to subjects with acute symptomatic encephalitis:

4) a higher frequency of 2DS4, 2DS5 and 3DS1 KIR genes

5) a higher frequency of 2DL2-HLA-C1 co-expression.

At multiple logistic regression analysis a detrimental effect towards acute symptomatic encephalitis of AA KIR haplotype and HLA-A-BW4and HLA-B-BW4^T^ alleles and of interaction between KIR 3DL1 and HLA-A-Bw4 alleles.

## MATERIALS AND METHODS

Between November 2014 and January 2016, all consecutive patients with symptomatic acute encephalitis were recruited from three wards (*Internal Medicine, Neurology, and Infectious Diseases*) of “P. Giaccone” University Hospital , Palermo, Italy. As healthy controls we enrolled *healthy blood donors*.

Encephalitis diagnosis was reached by a combination of clinical, laboratory, neuroimaging, and electrophysiologic findings according to well developed case definitions which generally require encephalopathy, as characterized by alteration in consciousness or personality change lasting for a sustained period of time (typically greater than 24 hours), presence of fever, cerebrospinal fluid (CSF) pleocytosis, or MRI or EEG changes compatible with encephalitis [15].

Type 2 diabetes mellitus was determined using a clinically based algorithm that considered age at onset, presenting weight and symptoms, family history, onset of insulin treatment, and history of ketoacidosis. Hypertension was defined according to the 2013 ESH/ESC guidelines [14].

### Exclusion criteria

For patients with acute encephalitis and subjects without acute encephalitis were: rheumatologic diseases, acute bacterial infections, other acute and chronic viral infections such as human immunodeficiency virus (HIV), *Hepatitis C virus (*HCV), hepatitis B virus (HBV) and acute infection with cytomegalovirus (CMV), Epstein barr virus (EBV) and recent acute cardiovascular and cerebrovascular events *(<1 month prior of admission)*.

### HLA and KIR genotyping

Informed consent was obtained for collection of samples from all patients or from their collaterals and controls. Consent forms were administered by physicians involved in the study.

Peripheral whole blood samples was collected as indicated, and genomic DNA was extracted from leukocytes by a commercial kit (PureLink^®^ Genomic DNA, ThermoFisher Scientific, Waltham, MA, USA).

The following KIR genes were analyzed: the inhibitory receptors KIR2DL1, 2DL2, 2DL3, 2DL5, 3DL1, 3DL2, 3DL3, the activating receptors 2DL4, 2DS1, 2DS2, 2DS3, 2DS4, 2DS5, 3DS1, 2 pseudogenes (2DP1 and 3DP1) and the common variants of KIR2DL5 (KIR2DL5A, KIR2DL5B), the KIR2DS4 alleles, and KIR3DP1 alleles. KIR gene profiles were determined by the presence or absence of each KIR gene.

Genotyping KIR and HLA ligands was performed using low resolution KIR-TYPE kit and Epitop Type strips . The KIR TYPE plates and Epitop-Type strips contained pre-dropped and dried reagents with allele specific primers, internal control primers (specific for the sequence on chromosome 1) and nucleotides.).

The detection of receptor Kir/HLA ligands was performed with the Sequence Specific Primer method (SSS PCR). HLA-C and Bw4 KIR ligand groups were assigned directly by using specific oligonucleotide primers to type the codon corresponding to amino acid 80 for HLA-C (HLA-C1, Cw alleles with asparagine at position 80; HLA-C2, Cw alleles with lysine at position 80) and for HLA-Bw4 (Bw4-I, Bw alleles with isoleucine at position 80; Bw4-T, Bw alleles with threonine at position 80).

### Statistical analysis

Statistical analysis of quantitative and qualitative data, including descriptive statistics, was performed for all items. Continuous data are expressed as mean ± SD, unless otherwise specified. Baseline differences between groups were assessed by the chi-square test or Fisher exact test, as needed for categorical variables, and by the independent Student *t* test for continuous parameters. Multinomial logistic regression analysis examined the correlation between patient characteristics (independent variables), and patient groups (dependent variable) in simple and multiple regression models. Data were analyzed by IBM SPSS Software 22 version (IBM Corp., Armonk, NY, USA). All p-values were two-sided and *p <* 0.05 was considered statistically significant.

## Abbreviations

HLA: human leukocyte antigen
HSE: Herpes simplex encephalitis
CNS: central nervous system
PRRs: pattern recognition receptors
NK: natural killer
CMV: cytomegalovirus
CSF: cerebrospinal fluid
MRI: magnetic resonance imaging
EEG: electroencephalogram
ESH/ESC: European Society of Hypertension/European Society of
HIV: human immunodeficiency virus
HCV: *Hepatitis C virus*
HBV: hepatitis B virus
SSS PCR: Sequence Specific Primer method
PCR: polymerase chain
WBCs: white blood cells
HSV-DNA: herpes simplex virus DNA
HHV type 6: Human *herpesvirus 6*
GvHD: graft versus host disease)
CD4: cluster of differentiation 4
CD8: cluster of differentiation 8
